# Irritable Bowel Syndrome (IBS) and Non-Celiac Gluten Sensitivity (NCGS): Where Is the Culprit Hiding? Nutritional Tips for Gastroenterologists

**DOI:** 10.3390/nu11102499

**Published:** 2019-10-17

**Authors:** Emanuele Rinninella, Marco Cintoni, Maria Cristina Mele, Antonio Gasbarrini

**Affiliations:** 1 UOC di Nutrizione Clinica, Dipartimento di Scienze Gastroenterologiche, Endocrino-Metaboliche Nefro-Urologiche, Fondazione Policlinico Universitario A. Gemelli IRCCS, 00168 Rome, Italy; 2 Istituto di Patologia Speciale Medica, Università Cattolica del Sacro Cuore, 00168 Rome, Italy; mariacristina.mele@unicatt.it (M.C.M.); antonio.gasbarrini@unicatt.it (A.G.); 3 Scuola di Specializzazione in Scienza dell’Alimentazione, Università di Roma Tor Vergata, Via Montpellier 1, 00133 Rome, Italy; marco.cintoni@gmail.com; 4 UOSA di Nutrizione Avanzata in Oncologia, Dipartimento di Scienze Gastroenterologiche, Endocrino-Metaboliche e Nefro-Urologiche, Fondazione Policlinico Universitario A. Gemelli IRCCS, Largo A. Gemelli 8, 00168 Rome, Italy; 5 UOC di Medicina Interna e Gastroenterologia, Dipartimento di Scienze Gastroenterologiche, Endocrino-Metaboliche e Nefro-Urologiche, Fondazione Policlinico Universitario A. Gemelli IRCCS, 00168 Rome, Italy

At least 40% of all the gastroenterological outpatient visits are due to functional gastrointestinal disorders (FGIDs), among which irritable bowel syndrome (IBS) is the most common, accounting for a worldwide prevalence of about 12% [1]. Patients affected by IBS have a significant impairment of their quality of life and productivity, and this, in turn, has an impact on social and economic costs [2].

The diagnosis of IBS lacks a specific biomarker and, to date, it relies on the so-called “Rome IV Criteria”, a symptom-based scheme requiring that patient complains of abdominal pain on average at least 1 day/week and that pain is associated with two or more of the following characteristics: (1) it is related to defecation; (2) it is associated with a change in the frequency of stool; or (3) it is associated with a change in the form (appearance) of the stool. These criteria should be fulfilled for the last 3 months, with the onset at least 6 months before diagnosis [3]. Although the physiopathology of IBS is not fully understood, a biopsychosocial model correlates early life’s sociocultural factors, family interactions, and psychosocial factors (i.e., abnormal emotional responses to stress and a sexual or physical abuse history) with functional gut alterations, such as abnormal motility, visceral hypersensitivity, immune dysregulation, inflammation, and barrier dysfunction, through the bidirectional neuroanatomic substrate of the “brain–gut axis” [3]. In some cases, the onset of the disease can appear after an infection, like acute bacterial, protozoal, or viral gastroenteritis [2]. Furthermore, a possible role of gut microbiota in the onset and maintenance of IBS was suggested [3]. Indeed, these patients, compared to the healthy controls, show a different gut microbiota composition, with the loss of microbial richness, an increased Firmicutes to Bacteroidetes ratio, and decreased *Lactobacillus* and *Bifidobacterium* abundances [4]. Even though up to 70% of patients experience symptoms after eating certain foods, like wheat or dairy products [5], a specific food or nutrient has not clearly been implicated in the pathogenesis of the disease. However, in the last ten years, a low fermentable oligo-, di-, and monosaccharide and polyol (FODMAP) diet, developed by the Monash University (Melbourne, Australia), was largely employed and was effective in reducing the symptoms of IBS patients [6]. FODMAPs are osmotically active short-chain carbohydrates, which are poorly absorbed and rapidly fermented by gut bacteria in the large intestine, thereby increasing intraluminal water volume by an osmotic effect and producing gas due to fermentation. To date, at least two meta-analyses have confirmed a significant reduction in abdominal pain and bloating in patients receiving a low-FODMAP diet compared with those receiving a standard diet, suggesting the key role of a low FODMAP diet in the first-line treatment of IBS [7,8]. 

Non-Celiac Gluten Sensitivity (NCGS) is a syndrome characterized by intestinal and extra-intestinal symptoms, related to the ingestion of gluten in subjects who are not affected by celiac disease (CD) or wheat allergy (WA) [9].

The prevalence of NCGS is still difficult to ascertain, ranging from 0.6% to 10.6% in the general population, according to studies. However, a lack of specific diagnostic tests (see below) suggests a selection bias [10]. As in IBS, abdominal pain, bloating, borborygmus, and changes in bowel habits may occur, and a specific diagnostic biomarker has not yet been identified. Unlike IBS, in NCGS, extra-intestinal symptoms such as a headache, dermatitis, and foggy mind can also be present. Gastroenterologists use the Salerno Experts’ Criteria to formulate the diagnosis of NCGS [9]. The Salerno diagnostic protocol uses a double-step approach defined only by symptoms. In Step 1, after excluding CD and WA through a clinical and laboratory evaluation, patients have to eat a gluten-containing diet for at least six weeks; then (at the baseline visit), they report symptoms according to a self-administered modified version of the Gastrointestinal Symptom Rating Scale (GSRS), grading them according to a Numerical Rating Scale (NRS). After that (time 0), they start a gluten-free diet (GFD) for at least six weeks, recording possible variations of the symptoms among those described above. A decrease of at least 30% of the baseline score is considered a positive response. Step 2 is called “the Gluten Challenge” and is required to confirm the diagnosis in patients responding to the GFD. This is provided through a reintroduction of gluten in a double-blind placebo-controlled challenge with cross-over design, in which a variation of symptoms of at least 30% between gluten and placebo discriminate a positive from a negative result [9]. 

Some concerns may afflict the Gastroenterologists in the clinical practice between IBS and NCGS. An interesting review published by Catassi et al. [10] tried to shed light upon this problem.

First, the lack of a specific diagnostic biomarker for the diagnosis of both IBS and NCGS can make the diagnostic approach difficult. This is especially true for NCGS, whose diagnostic protocol has been defined as “cumbersome” by the same experts of Salerno, and not apt for epidemiological studies [9].

Secondly, at present, a GFD is generally perceived by common people as “healthy”, and many individuals start a GFD just because they feel better, self-reporting an NCGS without a medical diagnosis and a personalized dietary plan. This issue also complicates the diagnostic protocol of NCGS, since patients have to eat a gluten-containing diet in Step 1 [10].

Thirdly, other wheat components may trigger an immune or inflammatory response, apart from gluten. For example, amylase trypsin inhibitors (ATIs), pest resistance molecules contained in the endosperm of wheat and related cereals, have been identified as strong activators of innate immune responses in human and murine macrophages, monocytes, and dendritic cells, eliciting the release of proinflammatory cytokines via the activation of toll-like receptor 4 (TLR4) [11]. In the same way, wheat germ agglutinins (WGA) have been shown to promote the release of pro-inflammatory cytokines, thus impairing the integrity of the intestinal epithelial layer [12]. Wheat contains also fructans—belonging to the category of FODMAPs—whose content varies according to the final product [13]. A recent double-blind placebo-controlled crossover study in patients with self-reported NCGS showed that fructans (rather than gluten) are more likely to induce symptoms, with no effect on the gluten challenge [14]. Another randomized clinical study reported that a double-blind gluten challenge induced symptom recurrence only in one-third of patients fulfilling the clinical diagnostic criteria for NCGS. Interestingly, in this trial, almost half of the patients, after the challenge, reported a recurrence of symptoms with gluten-free flour [15]. 

The differential diagnosis between IBS and NCGS is, furthermore, challenging since patients suspected to have IBS may undergo a low FODMAP diet, which excludes wheat due to its high content of fructans. In this way, gluten is avoided by default, without allowing for a possible diagnosis of NCGS. Indeed, patients tend to have rapid relief from gastrointestinal symptoms by avoiding FODMAPs, and physicians may often underestimate the role of gluten as the causative agent of disease. This common evidence has been already confirmed in a placebo-controlled, cross-over rechallenge study, in which the authors aimed to investigate the specific effect of gluten reintroduction after a low FODMAP diet in patients with self-reported NCGS. After a 2-week period of a low FODMAP diet, the authors found no significant gastrointestinal effects after gluten reintroduction through a high-gluten meal (16 g gluten/day) compared to a low-gluten meal (2 g gluten/day; 14 g whey protein/day) or a control meal (16 g whey protein/day) [16].

The huge impact of a low FODMAP diet on NCGS may cause confusion on at least two levels. (1) The appropriateness of diagnosis: Indeed, a population study (over 1000 patients) in the UK demonstrated that individuals with NCGS have a 20% prevalence of fulfilling the IBS diagnostic criteria [17]; (2) dietary management: Is it still recommended to prescribe a GFD to NCGS patients? Should the patients suspected to suffer from NCGS eat a low FODMAP diet?

On the other hand, a low FODMAP diet is not recommended as a long-term treatment even for IBS patients, given its significant impact on gut microbiota and because this impact has not yet been fully evaluated for its long-term clinical consequences [18]. 

Such evidence raises a question about the identification of gluten as the only culprit of the symptoms of NCGS. For these reasons, the term Non-Celiac Wheat Sensitivity (NCWS) appears, to date, more appropriate than NCGS to describe this syndrome [19]. 

In the above-cited review, Catassi et al. [10] proposed a practical guide for the Gastroenterologists, including the clinical assessment of IBS symptoms (Rome IV Criteria), the exclusion of alarm (red flag) features, and diagnostic tests to exclude other organic diseases. After that, first and second-line dietary recommendations are proposed, the latter according to diagnostic suspicions: (1) Consider trying a GFD for 4 to 6 weeks if the patient reports wheat and gluten-related intestinal and gastrointestinal symptoms, especially if anti-gliadin antibodies are present; (2) consider a low FODMAP diet if the patient self-reports predominantly gastrointestinal symptoms related to high FODMAP food. 

Finally, one must remember that “all wheat is not the same and all gluten is not the same” [10]. Recently, an Italian double-blind randomized cross-over trial [20] was released comparing the effects of an organic durum wheat variety (the ancient organic wheat single variety *Senatore Cappelli*) with those of standard commercial wheat varieties in patients with recognized NCGS, according to the Salerno Criteria. Enrolled patients experienced lower gastrointestinal and extra-intestinal symptom scores after eating the *Senatore Cappelli* wheat variety-based product than after eating standard commercial wheat products. This evidence, if confirmed in further studies, could open new dietary alternatives to GFD in NCGS with consequential health, economic, and social benefits.

In conclusion, FGIDs and NCGS may often overlap due to their common symptoms. A GFD and a low FODMAP diet are not always the only solution. Differential diagnosis requires both accurate diagnostic and therapeutic approaches, the latter mostly relying on nutritional counseling and a personalized dietary plan. Clinical Nutritionists, Dieticians, and Gastroenterologists should work together to better understand and manage such complex syndromes. 
